# Robot-assisted versus conventional minimally invasive surgery in the treatment of paediatric neuroblastoma: a single-centre retrospective study

**DOI:** 10.1007/s00383-026-06438-y

**Published:** 2026-04-21

**Authors:** Stefano Avanzini, Serena Reali, Federico Palo, Massimo Conte, Stefania Sorrentino, Filippo Spreafico, Girolamo Mattioli

**Affiliations:** 1 Paediatric Surgery Unit, IRCCS Istituto Giannina Gaslini, Via Gaslini 5, 16147 Genoa, Italy; 2 DINOGMI, University of Genova, Genoa, Italy; 3 Paediatric Oncology Unit, IRCCS Istituto Giannina Gaslini, Via Gaslini 5, 16147 Genoa, Italy

**Keywords:** Neuroblastic tumours, Neuroblastoma, Robot-assisted surgery, Minimally invasive surgery, IDRF, Paediatric oncology

## Abstract

**Introduction:**

Neuroblastoma is the most common extracranial solid tumour in childhood. Although minimally invasive surgery (MIS) is increasingly used in selected patients with neuroblastic tumours, comparative data between robot-assisted and conventional MIS are lacking.

**Materials and methods:**

A single-centre retrospective study was conducted on 84 patients who underwent minimally invasive resection of neuroblastic tumours between 2008 and 2025. Patients were divided into conventional MIS and robot-assisted surgery groups. Demographic data, tumour characteristics and surgical and oncological outcomes were compared.

**Results:**

The robot-assisted group included a higher number of patients with Image-Defined Risk Factor (IDRF) ≥ 1 (57% vs. 8%). Robotic surgery was therefore preferentially employed in more complex cases, resulting in higher conversion rate and longer operative time. Despite the higher prevalence of patients with IDRF ≥ 1 in the robotic group, intraoperative and postoperative complication rates were comparable between groups. Notably, conversions were not performed for emergency reasons but were based on intraoperative decision-making.

**Conclusions:**

Robot-assisted surgery may safely expand selection criteria to selected patients with positive IDRFs, while maintaining oncological principles and without increasing intra and postoperative morbidity.

**Supplementary Information:**

The online version contains supplementary material available at 10.1007/s00383-026-06438-y.

## Introduction

 Neuroblastoma (NB) is an embryonal tumour originating from immature sympathoadrenal precursor cells and represents the most common extracranial solid tumour in paediatric patients. The primary site of origin is the abdomen, with adrenal gland involvement in approximately 47% of cases and involvement of the abdominal sympathetic chain in 24%. Intrathoracic localization accounts for about 15% of cases, while cervical and pelvic localizations are rare, each occurring in approximately 3% of patients [1].

The laparoscopic approach in paediatric NB was first described by Yamamoto et al. in 1996 [2]. Since then, conventional minimally invasive surgery (MIS) has progressively gained acceptance in the treatment of neuroblastic tumours, leading to the publication of specific guidelines developed by International Study Groups, which define indications, selection criteria, and limitations of this approach [3–5]. The first case of robot-assisted resection of NB in paediatric patients was published by Meehan et al. in 2008 [6]. Despite the subsequent publication of case series [7–14] and studies with larger cohorts [15–20], the overall experience reported in the literature remains limited. Although several studies have described the use of conventional MIS or robotic-assisted surgery in the treatment of NB, it remains unclear whether robotic technology provides significant advantages over standard minimally invasive techniques. To date, only one study has compared the two surgical approaches in adrenalectomy for NB, but this study was limited to tumours confined to the adrenal gland and without the presence of Image-Defined Risk Factors (IDRFs) [21].

To our knowledge, this represents the largest single-centre series of minimally invasive resection of neuroblastic tumours published to date. Furthermore, the study presents a systematic comparison of clinical characteristics, surgical data, and outcomes between patients treated with conventional MIS and those undergoing robot-assisted procedure, with the aim of assessing whether robotic surgery provides an added value in the surgical management of neuroblastic tumours.

## Methods

Following approval of the study protocol by CER Liguria (477/2020), a single-centre retrospective study was conducted. All patients aged 0–18 years, with neuroblastic tumour who underwent surgical excision of the mass via MIS at Giannina Gaslini Institute were included. The study covered a period of 17 years (from October 2008 to November 2025). Patients who underwent minimally invasive surgical biopsy were excluded.

Robotic technology (Da Vinci XI Surgical System, Intuitive Surgical Inc.; Sunnyvale, California, United States) became available at our Centre for a limited period in 2016 and was systematically reintroduced starting from 2020. During periods when the robotic technology was unavailable, patients were treated using conventional MIS (laparoscopy or thoracoscopy). A dedicated database was established to collect the following data: patient demographic data (sex, age at surgery, weight, ASA – American Society of Anesthesiologists – class), tumour location, histopathological tumour characteristics (histology, MYCN amplification), INRGSS (International Neuroblastoma Risk Group Staging System) stage, preoperative IDRFs, tumour volume, preoperative medical therapy (chemotherapy, hematopoietic stem cell transplantation, radiotherapy, radiometabolic therapy), surgical approach, operative time and console time for robotic procedures, residual tumour, conversion to open surgery, intraoperative complications, blood losses, short-term postoperative complications (< 30 postoperative days), drain placement, length of hospital stay and oncological follow-up.

Postoperative complications were classified according to the standardized Clavien–Dindo classification system [22]. Tumour volume was reported both as ellipsoid tumour volume (ml), and as a percentage of the ratio between ellipsoid tumour volume (ETV) and the estimated patient blood volume (EPBV – calculated as 75 mL/kg for children older than three months), allowing a more accurate assessment of tumour size relative to patient age.

All patients underwent preoperative Magnetic Resonance Imaging (MRI) or Computed Tomography (CT) and were discussed by a multidisciplinary team with a paediatric surgeon, oncologist, and radiologist. The decision to pursue a minimally invasive rather than an open approach was made collectively during multidisciplinary tumour board discussions, based on imaging review, the patient’s clinical history and established MIS guidelines in paediatric oncologic surgery [3–5]. Eligibility for conventional MIS was limited to IDRF-negative cases, with rare exceptions. Following the introduction of robotic surgery at our Centre, and given the recognized advantages of this technique, selected IDRF-positive patients were considered eligible.

### Statistical analysis

Patients were divided into two groups: those undergoing conventional minimally invasive tumour resection (thoracoscopy or laparoscopy), and those treated with robot-assisted thoracoscopic or laparoscopic resection. Comparative statistical analyses were performed between the two groups. Data are presented as median and range (minimum - maximum values) for continuous variables and as absolute number and percentage for categorical variables. For continuous variables, after assessing normality using the Kolmogorov–Smirnov test, the Student’s t-test was used for normally distributed variables, and the Mann–Whitney U test was used for non-normal distributions. Categorical variables were compared using the χ² test, Fisher’s exact test, or the Fisher–Freeman–Halton test, as appropriate. A p-value < 0.05 was considered statistically significant.

## Results

The characteristics of the study population are summarised in Table 1 (demographic and preoperative data) and Table 3 (intraoperative and postoperative data). A total of 84 patients with neuroblastic tumours underwent MIS for tumour mass resection. Thirty-eight patients (45%) were treated with conventional MIS, while 46 patients (55%) underwent robotic-assisted surgery. The median age at surgery was 2 years and the median body weight was 13 kg. Tumour location was predominantly abdominal (73%), followed by thoracic (20%) and pelvic (7%). In four patients (5%), the resected lesion represented either a recurrence or a residual tumour, indicating a previous surgical intervention at the same site. At least one IDRF was present in 29 patients (35%). Several patients received preoperative medical treatment according to risk group protocols.

The median operative time was 123 min (range: 35–335 min). Conversion to open surgery was required in 11 cases (13%) and occurred more frequently in the robotic surgery group. Specifically, only one conversion was recorded in the conventional MIS group, due to IDRF positivity involving the subclavian vessels. In the robotic group, 10 conversions were recorded, eight of which were due to IDRF positivity involving vascular structures. In the remaining two cases, conversion was required because of intraoperative bleeding from the adrenal vein during adrenalectomy and to enable accurate lesion identification in a patient with residual disease following previous open resection of a thoracic NB. Notably, these two conversions occurred in patients without positive IDRFs. Residual tumour volume of less than 10% was documented in 26 patients (31%), while complete resection was achieved in the remaining cases; no residual tumour greater than 10% was recorded.

A total of three intraoperative complications (4%) were reported. In the conventional MIS group, a splenic injury occurred during trocar placement for thoracoscopic resection of a NB, necessitating emergency splenectomy. In the robotic surgery group, two complications were observed: one iatrogenic gallbladder injury requiring cholecystectomy in a patient with abdominal NB with three positive IDRFs, and renal ischemia in a patient with an abdominal tumour and a positive IDRF involving the renal hilum. In both cases, conversion to open surgery was required because of the risk associated with IDRF positivity.

Postoperative complications within 30 days occurred in 8 patients (10%). In the conventional MIS group, three infection-related complications were reported, all managed with antibiotic therapy. In the robotic surgery group, postoperative complications were observed in five patients. One patient developed ureteral stenosis following ureteral resection and anastomosis performed to ensure surgical radicality due to a positive IDRF for ureteral involvement; this complication was managed with ureteral stent placement. One patient required surgical revision on postoperative day 7 for bowel obstruction, with intraoperative findings of adhesive band and internal hernia. During the same hospital stay, this patient was also diagnosed with celiac axis thrombosis, managed with anticoagulation after unsuccessful local thrombolysis during arterial angiography, and with the development of an hypotrophic kidney secondary to intraoperative ischemia, being the tumour involving the renal pedicle. This was the only case in the entire cohort in which initiation of adjuvant therapy was delayed due to postoperative complications. Despite a complicated postoperative course, the child achieved complete recovery with no functional impairment of the splanchnic organs, although a hypotrophic kidney and celiac axis thrombosis were present. The patient is currently in complete oncologic remission. Additionally, two cases of trocar-site incisional hernia were recorded, both treated surgically, as well as one case of omental evisceration at the time of drain removal, resolved by bedside fascial closure under sedation. Detailed IDRFs, reasons for conversion to open surgery and intra and postoperative complications observed in the two groups are summarised in Table 3. The median length of hospital stay was 4 days (range: 1–30 days).

### Conventional MIS vs. robotic-assisted surgery

Table 1 also presents a comparison between patients undergoing conventional MIS and those undergoing robot-assisted surgery. Several statistically significant differences emerged between the two groups, despite comparable demographic and tumour characteristics. INRGSS staging differed significantly between the two cohorts, with a higher prevalence of stage L2 disease in patients treated with robot-assisted surgery. Similarly, the presence of at least one IDRF was significantly more frequent in the robotic group than in the conventional MIS group (57% vs. 8%; *p* < 0.001).

Tumour volume was greater in the robotic group compared with the conventional MIS group (16.65 vs. 4.85 ml; range: 0.4–376.8 vs. 0.4–123.8; *p* = 0.03). Likewise, the ETV/EPBV ratio was higher in the robotic group than in the conventional MIS group (1.64% vs. 0.41%; range: 0.04–21.20 vs. 0.04–33.01; *p* = 0.03).

Intraoperative data analysis showed a significantly longer operative time in patients undergoing robotic surgery (165 min; range: 50–335) compared with the conventional MIS group (90 min; range: 35–300), with p = < 0.001. A residual tumour volume < 10% was observed significantly more frequently in the robotic group than in the conventional MIS group (48% vs. 11%; p = < 0.001). Drain placement was significantly more frequent in robot-assisted patients (67% vs. 21%; p = < 0.001). No significant differences were found between the two groups regarding intra and postoperative complications within 30 days. The length of hospital stay was comparable between the two groups with a median of 4 days. However, among patients who were not converted to open surgery and who completed the procedure using a minimally invasive approach, the median length of stay was significantly shorter in the robot-assisted group with a median of 3 days (range: 2–7), compared with 4 days (range: 1–24) in the conventional MIS group (*p* = 0.004).

## Discussion

Open surgery has historically been considered the gold standard for the surgical treatment of neuroblastic tumours. However, advancements in surgical technique have progressively expanded the role of MIS as a feasible approach in selected patients, while maintaining strict adherence to oncological principles [23, 24]. Several studies compared open surgery and conventional MIS for neuroblastic tumours [25–30]. In addition to cosmetic benefits, MIS has demonstrated significant advantages, including reduced intraoperative blood losses [26, 27, 30], faster postoperative recovery with a shorter length of hospital stay [25–28], and a shorter interval to initiation of adjuvant chemotherapy [25], while providing oncological outcomes comparable to those of open surgery [26–30]. Considering the growing experience in minimally invasive techniques, several International Study Groups have proposed specific guidelines for MIS treatment in neuroblastic tumours, with the aim of defining selection criteria for this approach [3–5]. The main recommendations proposed by these guidelines are summarized in Supplemental Table 1. With the advent of robotic surgery and its recognized advantages, such as high-definition 3D vision, articulated movements with seven degrees of freedom, tremor filtration, and improved surgeon ergonomics, its use has progressively increased in paediatric oncological surgery, including the treatment of neuroblastic tumours [18, 19]. Supplemental Table 2 summarizes the available literature on robotic surgery for neuroblastic tumours. To date, the evidence and existing guidelines are limited to single-centre experiences and are therefore largely dependent on individual surgeons’ skills and expertise. However, only one retrospective study has systematically evaluated the benefits of robotic surgery for adrenalectomy in patients with localized neuroblastoma without IDRFs and demonstrated an advantage of the robotic approach in terms of reduced intraoperative blood losses and shorter hospital stay [21].

The present study reports the largest single-centre series of patients with neuroblastic tumours treated with MIS, providing a detailed analysis of demographic characteristics, intraoperative data, and surgical and oncological outcomes. Moreover, this is the first study to compare conventional MIS and robotic surgery for neuroblastic tumours resection, including all anatomical sites. In our experience, both minimally invasive approaches proved to be feasible and safe even in fragile and highly complex patients, including those with increased anaesthetic risk and those previously treated with neoadjuvant medical therapy. In addition, both techniques were effective in the management of recurrent and residual neuroblastic tumours, suggesting that these conditions should not be considered contraindications to MIS.

A significant difference in IDRF positivity was observed between the two groups. Among patients who did not require conversion, only two patients in the conventional MIS group had at least one IDRF. Conversely, in the robotic-assisted group, 18 patients presenting with at least one IDRF, successfully completed the procedure minimally invasively without intraoperative complications. These results, although encouraging, must be interpreted considering the retrospective nature of the study and the inherent selection bias. At our Centre, conventional MIS was generally reserved for IDRF-negative neuroblastic tumours, with only exceptional cases considered eligible [31]. These criteria are consistent with the available literature [29–35] and with International Study Groups guidelines [3–5]. Following the introduction of robotic technology at our Institution and considering its recognized advantages, including improved delineation of tumour extent and greater precision in dissecting vascular structures and adjacent organs frequently involved by the tumour [7, 17, 19], its use was progressively expanded. Based on this and supported by experiences reported from other paediatric Centres [11, 15], it was deemed appropriate to progressively broaden selection criteria to include patients with positive IDRFs. Therefore, despite the selection bias between the two groups, our findings suggest that robotic surgery was particularly advantageous in our setting, enabling successful management of a significantly higher number of cases with positive IDRFs.

Conversion rate was higher in robot assisted group compared to conventional MIS group. Except for one case requiring urgent conversion for intraoperative bleeding from the adrenal vein, the remaining conversions were planned intraoperatively before potential complications occurred. These conversions were prompted by the presence of IDRFs that could not be safely managed using a minimally invasive approach because of technically challenging dissection and the need to ensure adequate oncological radicality. Although MIS carries an inherent risk of conversion, it proved to be safe when performed by an experienced oncologic surgeon capable of recognizing the limitations of the approach and adopting the most appropriate strategy to ensure patient safety and optimal oncological outcomes. The conversion rate for robotic neuroblastoma surgery reported in the two largest series in the literature is 2% [18, 19]. However, in one of these studies, the percentage of patients with at least one positive IDRF was only 4% [18], while in the other it was 33% [19], both lower than the 57% observed in our population, which was associated with a conversion rate of 22%. As previously demonstrated, not all IDRFs carry the same weight in terms of surgical risk [36, 37], and they differentially influence the likelihood of conversion. In our experience, two patients in the conventional MIS group presenting with IDRFs safely underwent totally minimally invasive resection; however, in both cases, the procedure required sacrifice of the structure involved by the IDRF. Specifically, one patient with encasement of the internal iliac artery required vascular ligation, while another with pulmonary infiltration underwent atypical lung resection. The only patient with a positive IDRF involving the subclavian vessels required conversion due to the inability to safely complete the procedure thoracoscopically. In the robotic group, among 26 patients presenting with at least one IDRF, only eight required conversion to open surgery. Of these, four had encasement of the renal pedicle and one had encasement of the iliac vessels. Notably, three out of four patients presenting with multiple IDRFs required conversion to an open approach, suggesting that clusters of abdominal IDRFs are associated with a higher risk of conversion. Furthermore, it is important to highlight that patients with high-risk IDRFs, such as infiltration of the hepatoduodenal ligament and the duodeno-pancreatic block, were successfully managed using a robotic approach. This underscores the need for further studies analysing the specific impact of individual IDRFs on conversion risk to improve patient selection for minimally invasive approaches.

The incidence of minimal residual tumour (< 10%) was higher in patients treated with robotic surgery. This result seems not to be correlated to the technique itself, but rather to the greater number of patients with positive IDRFs in the robotic group. Indeed, IDRF positivity is known to be associated with lower rates of complete resection and increased risk of postoperative complications [38, 39]. Nevertheless, oncological principles were respected in all cases, in fact surgery in neuroblastoma treatment aims to achieve local disease control through Gross Total Resection (GTR) or at least 90% resection of the primary tumour while minimizing morbidity [40, 41].

Despite the higher number of patients with positive IDRFs in the robotic group, the frequency of intra and postoperative complications was comparable between the two groups. In the robotic group, two cases of trocar-site incisional hernia and one case of omental evisceration during drain removal were observed. These complications were likely related to the larger size of the trocars used (robotic: 8 mm, conventional MIS: 5 mm except at the umbilicus). These events highlight the importance of meticulous abdominal wall closure, particularly when a drain is placed through a trocar site; in such cases, the fascial defect should be carefully closed around the drain to minimize the risk of herniation or evisceration after removal. Additionally, oncologic patients may represent a higher-risk population due to impaired wound healing from concomitant systemic therapies and poor nutritional status, emphasizing the need for careful operative technique and postoperative monitoring.

In our cohort, tumour volumes in the conventional MIS group were in line with literature recommendations (IPEG [3] and APSA [4]: tumour size < 6 cm; SIOPEN [5]: tumour volume < 75 ml, or < 60 ml with IDRF positivity), with only two patients presenting a tumour volume > 75 ml. For robot-assisted neuroblastic tumour resection, a selection criterion based on the ratio between tumour volume and estimated patient blood volume (ETV/EBV) has been proposed, identifying a value > 2% as a formal contraindication to robot-assisted surgery [19]. However, in our experience we successfully treated 25 patients with ETV/EBV > 2% (9 in the conventional MIS group and 16 in the robotic group) without conversion. Among these, 20 patients had an ETV/EBV between 2% and 10%, while five had values exceeding 10%. In agreement with the findings reported by Chang et al. [18], we believe that tumour volume itself is a less decisive selection criterion for patients eligible for robotic surgery and that the ETV/EBV > 2% cutoff should be further evaluated through multicentre studies and larger series.

Total operative time was longer in the robotic group compared with the conventional MIS group. This difference is likely attributable to the greater complexity of cases in the robotic cohort, which included a higher number of IDRF-positive patients, as well as a higher conversion rate to open surgery, reflecting the more challenging nature of these cases. Notably, console time in the robotic group was comparable to the operative time of conventional MIS. Although operative time for robotic surgery was longer than that for conventional MIS, it was comparable or shorter than the operative times reported in the literature [15, 18].

Despite the greater complexity of cases in the robotic group, overall postoperative length of stay was similar between robotic surgery and conventional MIS. When considering only patients who completed the procedure entirely via a minimally invasive approach without conversion to open, the robotic approach was associated with a shorter hospital stay in our experience, with a median of 3 days compared to 4 days in the conventional MIS group, consistent with the findings reported by Liu et al. [21]. This highlights that, when procedures are fully completed minimally invasively, the robotic approach may offer an advantage in terms of hospital stay. The mortality rate during oncological follow-up was higher in the conventional MIS group than in the robotic group. However, differences in oncological outcomes are likely influenced by improved survival rate in patients with NB due to the introduction of new therapeutic protocols [42], and clearly depend on intrinsic tumour biological and genetic characteristics that significantly affect outcomes. Additionally, follow-up in the robotic group is shorter, with the last patient having only 3 months of follow-up, leaving a residual risk of disease progression or recurrence. In contrast, follow-up in the conventional MIS group extends to at least 6 years.

This study is limited by its retrospective design and the lack of randomization, with an inherent risk of selection bias, making the two groups not fully comparable. In our cohort, patients presenting with at least one IDRF were more frequently allocated to the robotic-assisted approach, making direct comparison challenging. In theory, a prospective randomized study would be required to definitively overcome this limitation. However, this is unlikely to be feasible in clinical practice, as current MIS guidelines recommend strict selection criteria, making random allocation ethically and practically challenging. Moreover, although the available experience with robotic surgery remains limited, emerging evidence suggests that, in carefully selected cases, robotic technology may safely extend the boundaries beyond the traditional indications established for conventional MIS. Therefore, these results should be interpreted with caution, taking into account the methodological and clinical constraints inherent to the study design.

## Conclusions

This study supports the safety and feasibility of MIS in the treatment of neuroblastic tumours in selected patients. Our experience with conventional MIS has naturally progressed toward the use of robotic-assisted surgery, which, thanks to its technical advantages, appears to allow minimally invasive treatment in carefully selected patients with positive IDRFs, without increasing intra or postoperative morbidity and with adherence to oncological principles of resection. While clear guidelines are already available for conventional MIS, further work is needed to establish appropriate inclusion criteria for robotic procedures. The retrospective design and patient selection bias should be considered when interpreting these results. Nevertheless, the data support the role of robotic surgery as a valid advancement of minimally invasive techniques in the treatment of neuroblastic tumours. Further multicentre studies are needed to better define which patients are most likely to benefit from this approach.

**Table 1:** Study population characteristics and preoperative comparison between the conventional minimally invasive resection group and the robotic resection group

	TOTAL (*n* = 84)	Conventional MIS (*n* = 38)	Robotic surgery (*n* = 46)	*p*-value
F	45 (54%)	20 (53%)	25 (54%)	0.88
M	39 (46%)	18 (47%)	21 (46%)	
Age (years)	2 (0–18)	1 (0–18)	3 (0–15)	0.32
Weight (kg)	13 (5–95)	12 (5–75)	13.5 (5.8–95)	0.70
ASA:				0.002*
- ASA I	9 (11%)	8 (21%)	1 (2%)	
- ASA II	46 (55%)	23 (61%)	23 (50%)	
- ASA III	29 (34%)	7 (18%)	22 (48%)	
Tumour location:				0.29
- Abdominal	61 (73%)	30 (79%)	31 (67%)	
- Pelvic	6 (7%)	1 (3%)	5 (11%)	
- Thoracic	17 (20%)	7 (18%)	10 (22%)	
Previous surgery	4 (5%)	1 (3%)	3 (7%)	0.62
Histology:				0.87
- NB	63 (75%)	30 (80%)	33 (72%)	
- GNBn	1 (1%)	0 (0%)	1 (2%)	
- GNBi	11 (13%)	4 (10%)	7 (15%)	
- GN	9 (11%)	4 (10%)	5 (11%)	
MYCN amplification	7 (8%)	2 (5%)	5 (11%)	0.45
MYCN gain	6 (7%)	2 (5%)	4 (9%)	0.69
INRGSS:				< 0.001*
- L1	37 (44%)	22 (58%)	15 (33%)	
- L2	19 (23%)	2 (5%)	17 (37%)	
- M	22 (26%)	9 (24%)	13 (28%)	
- MS	6 (7%)	5 (13%)	1 (2%)	
IDRF:				< 0.001*
- 0 IDRF	55 (65%)	35 (92%)	20 (43%)	
- 1 IDRF	24 (29%)	2 (5%)	22 (48%)	
- 2 IDRF	4 (5%)	1 (3%)	3 (7%)	
- 3 IDRF	1 (1%)	0 (0%)	1 (2%)	
IDRF ≥ 1	29 (35%)	3 (8%)	26 (57%)	< 0.001*
Tumour volume (ml)	11.7 (0.4-376.8)	4.85 (0.4-123.8)	16.65 (0.4-376.8)	0.03*
ETV/EPBV (%)	0.79 (0.37–33.01)	0.41 (0.04–33.01)	1.64 (0.04–21.20)	0.03*
Preoperative medical treatment:				
- Chemotherapy	38 (45%)	12 (32%)	26 (57%)	0.02*
- HSCT	16 (19%)	8 (21%)	8 (17%)	0.67
- Radiotherapy	2 (2%)	2 (5%)	0 (0%)	0.20
- Radiometabolic therapy	2 (2%)	1 (3%)	1 (2%)	1

*Statistically significant values

MIS: minimally invasive surgery; F: female; M: male; ASA: American Society of Anesthesiologists; NB: neuroblastoma; GNBn: ganglioneuroblastoma nodular; GNBi: ganglioneuroblastoma intermixed; GN: ganglioneuroma; INRGSS: International Neuroblastoma Risk Group Staging System; IDRF: Image-Defined Risk Factor; ETV: ellipsoid tumour volume; EPBV: estimated patient blood volume; HSCT: Haematopoietic Stem Cell Transplantation

**Table 2:** Study population characteristics and intraoperative and postoperative comparison between the conventional minimally invasive resection group and the robotic resection group

	TOTAL (*n* = 84)	Conventional MIS (*n* = 38)	Robotic surgery (*n* = 46)	*p*-value
Surgical time (minutes)	123 (35–335)	90 (35–300)	165 (50–335) Console: 93 (23–195) - *R*	< 0.001*
Conversion to open surgery	11 (13%)	1 (3%)	10 (22%)	0.01*
Intraoperative complications	3 (4%)	1 (3%)	2 (4%)	1
Minimal residual tumour (< 10%)	26 (31%)	4 (11%)	22 (48%)	< 0.001*
Drain	39 (46%)	8 (21%)	31 (67%)	< 0.001*
Drain (days)	3 (1–9)	3.5 (2–5)	3 (1–9)	0.24
Postoperative complications (< 30 days)	8 (10%)	3 (8%)	5 (11%)	0.72
Length of hospital stay (days)	4 (1–30)	4 (1–24)	4 (2–30)	0.12
Oncologic follow-up: - Complete remission - Stable residual tumour - Disease progression - Death	65 (77%) 9 (11%) 1 (1%) 9 (11%)	28 (74%) 2 (5%) 0 (0%) 8 (21%)	37 (81%) 7 (15%) 1 (2%) 1 (2%)	0.01*

**Table 3:** Detailed IDRFs, reasons for conversion to open surgery and intra and postoperative complications observed in the two groups

Conventional MIS	Robotic surgery
**IDRFs**	
Tumour encasing subclavian vessels: 1# Tumour encasing the iliac vessels + more than 1/3 of the spinal canal invaded: 1 Infiltration of other organ (Lung): 1	Lower mediastinal tumour infiltrating the costo-vertebral junction between T9 and T12: 4 Tumour infiltrating the hepatoduodenal ligament: 1 Tumour invading one renal pedicle: 9 (4#) Tumour encasing the iliac vessels: 2 (1#) Tumour crossing the sciatic notch: 1 More than 1/3 of the spinal canal invaded: 3 Infiltration of duodeno-pancreatic block: 1 Infiltration of other organ (Ureter): 1 Tumour invading one renal pedicle + encasing the aorta and/or vena cava: 2 (1#) Tumour invading one renal pedicle + infiltration of kidney: 1# Tumour encasing branches of the superior mesenteric artery at the mesenteric root + invading one renal pedicle + encasing the aorta and vena cava: 1#
**Conversion to open surgery**	
Blood vessels encasement/adherence: 1	Blood vessels encasement/adherence: 8 Intraoperative bleeding: 1 Tumour identification: 1
**Intraoperative complications**	
Iatrogenic splenic injury (trocar) – splenectomy: 1	Iatrogenic gallbladder injury – cholecystectomy: 1# Renal ischemia: 1#
**Postoperative complications (< 30 days)**	
Infection-related complications – antibiotic therapy: 3 (CD: II)	Ureteral stricture – stent: 1 (CD: IIIb)
	Small bowel obstruction + celiac axis thrombosis + atrophic kidney – adhesiolisis + anticoagulant therapy: 1# (CD: IIIb)
	Trocar-site incisional hernia – hernia repair: 2 (CD: IIIb)
	Omental evisceration (following drain removal) – bedside fascial closure under sedation.: 1 (CD: IIIa)

IDRFs: Image Defined Risk Factors; # Converted patients; CD: Clavien-Dindo

## Supplementary Information

Below is the link to the electronic supplementary material.

Supplementary file1

## Data Availability

The data that support the findings of this study are available from the corresponding author S.R. upon reasonable request.
