# Supplementary material for: Robot-assisted versus conventional minimally invasive surgery in the treatment of paediatric neuroblastoma: a single-centre retrospective study
Source: Pediatr Surg Int. 2026 Apr 21;42(1):200. doi: 10.1007/s00383-026-06438-y (PMC13099698; doi:10.1007/s00383-026-06438-y)
Supplement: Supplementary file 1 — Supplementary file1 [file 383_2026_6438_MOESM1_ESM.docx]

**Supplemental Table 1.** Recommendations from International Study Groups on minimally invasive surgery for neuroblastic tumours.

| **Group** | **Recommendations** | **Contraindications** |
| --- | --- | --- |
| **IPEG - International Pediatric**  **Endosurgery Group (2010)** [3] | Laparoscopic biopsy: to be considered in advanced neuroblastoma  Laparoscopic adrenalectomy: to be considered for small early tumours without evidence of invasion on preoperative imaging; may also be considered if the neuroblastoma responds to chemotherapy  Adherence to oncologic principles  Use of an endobag for tumour extraction | ***Relative contraindications*:**   - Tumour size > 6 cm - Enlarged veins - Involvement of adjacent organs or vessels |
| **APSA - American Pediatric Surgical**  **Association (2020)** [4] | Laparoscopic resection: safe for small abdominal tumours (4–6 cm)  Thoracoscopic resection: tumour size is a less well-defined criterion  Adherence to oncologic principles | IDRF-positive |
| **SIOPEN - International Society of Paediatric Oncology European Neuroblastoma Research Network** **(2022)** [5] | Minimally invasive resection: should be considered for small, localized, IDRF-negative neuroblastic tumours and with tumour volume <75 ml  Multidisciplinary discussion for MIS indication  Thoracic neuroblastic tumours: should be approached with caution, as operative complications are more frequent in these patients  Intraoperative findings must be documented and reported according to the “International Neuroblastoma Surgical Report Form (INSRF)[32]” | IDRF-positive  (*Relative contraindications*)  Presence of more than one IDRF, especially when associated with invasion of organs and vessels and/or tumor volume >60 ml |

**Supplemental Table 2.** Review of the literature on robotic surgical management of neuroblastic tumours.

| **Author** | **Number of tumours** | **Age** | **Histology** | **Tumour location** | **Tumour size** | **IDRF** | **Surgical time** | **Conversion** |
| --- | --- | --- | --- | --- | --- | --- | --- | --- |
| Meehan et al (2008)[6] | 2 | 2 – 4 years | GNB (1)  GN (1) | Thoracic (2) | 6.5 x 4.2 x 1.7 cm  4 x 2 x 2.5 cm | 0 | 69 – 44 min | 0 |
| Meehan et al (2013)[7] | 6 | - | NB (3)  GNB (1)  GN (2) | Abdominal (4)  Thoracic (2) | - | - | - | 2 |
| Uwaydah et al (2014)[8] | 1 | 15 months | NB | Abdominal | 4 x 2.8 x 2 cm | 0 | - | 0 |
| Meignan et al (2018)[9] | 3 | 0.75 – 1.17 – 1 year | NB (3) | Abdominal (3) | 2.6 x 2 x 2 cm  3.4 x 2.3 x 1.9 cm  2.5 x 1.5 x 1.5 cm | 0 | 115 – 93 – 105 min | 0 |
| Varda et al (2018)[10] | 1 | 7 years | GN | Abdominal | 6.4 cm | 0 | 172 min | 0 |
| Chen et al (2019)[11] | 1 | 3 years | NB | Abdominal | - | 2 IDRF | 389 min | 0 |
| Mitra et al (2020)[12] | 2 | 2 – 4 years | GNB (2) | Abdominal (2) | 2.5 cm - 6.4 cm | 0 | 244 – 265 min | 0 |
| Blanc et al (2022)[15] | 31 | - | NB (18)  GNB (4)  GN (9) | Abdominal (17)  Thoracic (10)  Pelvic (4) | ETV/EPBV: 0.2% (0.1–0.5) | 1 IDRF (9)  2 IDRF (1) | 215 min (156–282) | 2 |
| Vatta et al (2022)[13] | 2 | 7 – 9 years | NB (1)  GNB (1) | Abdominal (1)  Thoracic (1) | - | 0 | 320 min – 290 min | 0 |
| Ochi et al (2023)[14] | 1 | 35 months | NB | Thoracic | 5 cm | 0 | - | 0 |
| Palo et al (2024)[16] | 24 | - | NB (19)  GNB (2)  GN (3) | Abdominal (13)  Thoracic (6)  Pelvic (2) | ETV/EPBV: 0.15% (0.01–1.15) | 1 IDRF (7)  2 IDRF (1)  3 IDRF (1) | - | 8 |
| Taghavi et al (2024)[17] | 23 | 3 years (2-5) | NB (20)  GNB (1)  GN (2) | Abdominal (23) | 2.9 cm (2.5–4.8) | 1 IDRF (8) | 130 min (83-178) | 0 |
| Chang et al (2025)[18] | 49 | 49 months (22.5-86.5) | NB (16)  GNB (26)  GN (7) | Abdominal (46)  Pelvic (3) | 4.6 cm  (the largest: 8.7 cm) | 1 IDRF (4) | 205 min (165-304.5) | 1 |
| Blanc et al (2025)[19] | 51 | 3 years (0.8-18) | NB (33)  GNB (10)  GN (8) | Abdominal (31)  Thoracic (10)  Pelvic (5)  Other (5) | - | ≥ 1 IDRF (17) | - | 1 |
| Palo et al (2025)[20] | 10 | 57 months (16-175) | NB (5)  GNB (2)  GN (3) | Thoracic (10) | - | - | 153 min (65-240) | 1 |
| Liu et al (2025)[21] | 10 | 121.40 ± 51.27 months | NB (10) | Abdominal (10) | 3. 6 cm ± 1.3 | 0 | 177.5 min (147.3-280.75) | 0 |

NB: neuroblastoma; GNB: ganglioneuroblastoma; GN: ganglioneuroma; ETV: ellipsoid tumour volume; EPBV: estimated patient blood volume; IDRF: Image-Defined Risk Factor.
